# For the love of frontier research, or why Elon’s rockets keep blowing up

**DOI:** 10.1038/s44319-025-00587-2

**Published:** 2025-09-29

**Authors:** Nektarios Tavernarakis

**Affiliations:** 1 Department of Basic Sciences, School of Medicine, University of Crete, Heraklion, Crete 71003 Greece; 2 Institute of Molecular Biology and Biotechnology, Foundation for Research and Technology-Hellas, Heraklion, Crete 70013 Greece

**Keywords:** Economics, Law & Politics, History & Philosophy of Science, Science Policy & Publishing

## Abstract

The current Zeitgeist favouring utilitarian research risks undermining basic research—the very foundation on which innovative technological advances depend.

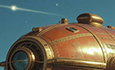

While undeniably bold, the SpaceX Starship program with its frequent spectacular failures epitomizes the perils of the current shift towards an applied-first innovation ethos that marginalizes foundational science. Interplanetary or interstellar space travel will likely not yield to the ostentatious attitude of “moving fast, breaking things”, and iterating on the road to success. If humanity ever reaches Mars, it may not be with a bigger rocket, but with means derived from entirely new principles, the kind that emerge only when we choose to understand the physical world through basic scientific inquiry. This becomes even more relevant for tackling urgent global challenges including the climate crisis, biodiversity loss, emerging diseases, resource depletion, food security or poverty.

Basic research, often termed fundamental, frontier, blue-sky, curiosity-driven—or even useless—is the pursuit of knowledge for its own sake, unshackled by immediate practical applications (Flexner, 2017). It is the bedrock of scientific progress, ushering in discoveries that reshape our understanding of the universe and fuel technological innovation. Yet, the current *Zeitgeist*, particularly in the USA and Europe, favors utilitarian, applied research, explicitly concocted on the premise of generating measurable outcomes with clear and immediate economic returns or social impact. While superficially appealing to certain funding bodies and politicians operating within short electoral cycles, this approach represents a fundamental misunderstanding of how the processes of scientific discovery and technological advancement actually work. The ongoing deliberations about the next EU Multiannual Financial Framework for 2028–2034 will be a litmus test of whether European policymakers are willing to confront this short-sightedness. Innovation does not occur in a vacuum. It requires raw materials: the findings of basic research. We are currently burning through a stockpile of knowledge accumulated during a more enlightened era of public investment, while allocating insufficient resources for replenishing it. To accomplish this critical replenishment does not mean abandoning applied research or ignoring practical applications. It means recognizing that fundamental science and technology development exist in a symbiotic relationship that requires both components to effectively facilitate disruptive innovation.

We are currently burning through a stockpile of knowledge accumulated during a more enlightened era of public investment, while allocating insufficient resources for replenishing it.

## The ‘*end of history*’ delusion

The recent shift towards prioritizing “gold standard”, mainly applied research at the expense of fundamental, curiosity-driven science, often cloaked in the rhetoric of efficiency, innovation, fiscal responsibility, and economic competitiveness, is deeply short-sighted. It conflates ephemeral deliverables with genuine innovation and confuses the act of producing with the act of discovering. As a result, it risks undermining the very foundations upon which modern civilizations have been built. This policy reorientation represents a profound failure of vision, both scientific and political, a capitulation to a culture that has lost patience with the long arc of frontier research which, however, does not bent to ambition alone. It exposes a dangerous form of technocratic exceptionalism, the belief that our current knowledge and paradigms are so complete, we can now abandon the very processes that generated them.

Indeed, we live in an era that flatters itself with a false sense of completeness, a new form of the “end of history” delusion applied to science. This perspective assumes that we are approaching the limits of knowledge itself, and that from here on, the task is one of deployment rather than of discovery. In such a worldview, basic science appears indulgent, even decadent, and applied research becomes the moral imperative. But history teaches us otherwise. The most transformative technologies, from quantum mechanics applications to CRISPR gene editing, emerged not from goal-directed efforts but from foundational inquiries with no predetermined outcomes. The gravest error of contemporary science policy is to assume that such outcomes can be summoned on demand, as though innovation were a vending machine fueled by targeted investment rather than by the slow, uncertain and often serendipitous crawl of basic research.

The most transformative technologies […] emerged not from goal-directed efforts, but from foundational inquiries with no predetermined outcomes.

## The perilous path of utilitarian myopia

This policy shift is also symptomatic of a broader cultural condition: the triumph of ‘instant gratification’ over long-term vision. In a political culture driven by election cycles and quarterly reports, investments in basic research, whose dividends may payoff decades in the future, are simply too slow to serve as political capital. Yet, this impatience betrays the responsibilities of governance. States and governments should not mirror the short-termism of markets; they should temper it. They must act, as Vannevar Bush argued, with a vision that transcends commercial or electoral calculus. His landmark report *Science, The Endless Frontier* laid the groundwork for America’s post-war scientific dominance, by asserting that government had a moral and strategic obligation to fund basic science, precisely because no one else would (Bush, 1945). To abandon that principle now, just as other global powers double down on state-led science strategies, is an act of strategic amnesia. Instead, democracy demands leaders with wisdom and vision beyond electoral horizons, capable of funding science that outlasts them.

Similarly, myopic views infect the education sphere, where the discussion about “entrepreneurial universities” signals the capitulation of higher education to market logic. Under the guise of employability and relevance, we are outsourcing curricula to the labor market, sacrificing the deep intellectual training that comes with engagement in basic science. The imposition of market logics, the commodification of education and the expansion of managerialism, are progressively turning universities into corporate bureaucracies, preoccupied less with the advancement of knowledge than with metrics, rankings, revenue accumulation, and risk management. Universities are not and were never meant to be vocational training centres. They are crucibles of thought, intellectual freedom, and experimentation. When their funding becomes dominated by top-down, mission-driven calls, it skews priorities toward safe bets and shifts their role from cultivating knowledge to servicing short-term tech goals and prescribed pay-offs. By emphasizing only what is immediately useful, at the expense of hypothesis-driven, investigator-led research, we rob the next generation of the tools they need to ask the right questions, let alone answer them.

The disconcerting trend of subordinating universities to partisan agendas was recently laid bare by the incendiary statements of prominent US officials, who portray academic institutions and their professors, in Nixon’s terms, as “the enemy”. This inflammatory rhetoric effectively positions evidence-based inquiry and academic discourse as threats to political authority rather than as essential components of informed governance. It delegitimizes the expertise of scholars, restricts funding streams, and promotes legislation that threatens the independence of research institutions. The targeting of universities also signifies a direct assault on the peer review system that ensures scientific quality and reliability. Academic institutions provide the infrastructure for rigorous evaluation of research claims, replication of studies, and the gradual accumulation of reliable knowledge. The slander of academia not only undermines public trust in credible institutions, it also fuels incredulity towards science and erodes the conditions necessary for maintaining a robust, open and forward-looking research ecosystem. Scientists may increasingly self-censor, avoiding topics or conclusions that might be perceived as politically inconvenient. Graduate students and early-career scholars may redirect their efforts away from research areas that could invite government scrutiny. The long-term consequence is an environment where political prejudice, rather than empirical evidence, shape research agendas and scholarly discourse. Discounting expertise in favor of ideological purity is a clear hallmark of authoritarian regimes.

## A steampunk dystopia

**Figure 1:**
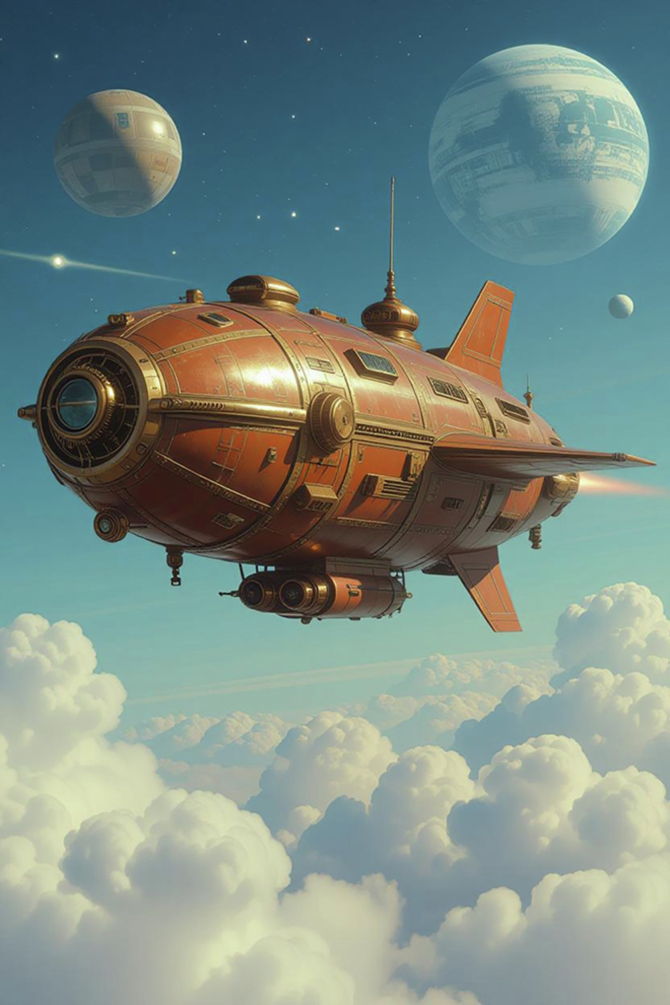
‘Steampunk’ space travel. Without basic research, technological development is limited to existing knowledge.

These tectonic mindset swings are compounded by an increasingly narrow imagination. Today’s policy frameworks often imagine the future using only the tools and ideas already available: a ‘steampunk mentality’ and a vision of progress that repurposes present know-how into slightly shinier iterations (Fig. 1). History shows that revolutionary technologies emerge only when researchers break free from existing notions of what is possible. Focusing only on marketable improvements means we will increasingly hit the wall of what can be done without new basic knowledge.

A helpful metaphor comes from the history of electric lighting. As Oren Harari remarked, the incandescent lamp did not emerge from “the continuous improvement of candles” (Beck and Charitos, 2021). In the late 19th century, one could contemplate highly sophisticated candle technology, self-trimming wicks, slow-burning waxes, and ornate fixtures that would represent the pinnacle of applied research in illumination. Yet, without Michael Faraday’s seemingly “useless” experiments on electromagnetic induction, and James Clerk Maxwell’s abstract mathematical formulation of electromagnetic theory, the electric light bulb would not have been possible. If we underfund blue-skies research, we will end up lighting elaborate candles in the dark, mistaking the flicker for real illumination. The result is stagnation and iteration disguised as innovation. Without basic research to expand our conceptual toolkit, we begin to exhaust what is possible with current paradigms. Indeed, major leaps – from candles to lightbulbs to today’s LEDs – depended on insights far removed from contemporary engineering goals. True innovation often requires stepping outside these current paradigms, not refining them.

The current fetish for top-down mega projects only exacerbates this problem. Such projects often promise inflated deliverables to justify their scale but struggle to produce the kind of conceptual breakthroughs that smaller, curiosity-driven inquiries have historically delivered. Whether in space research, in energy or in biomedical engineering, the history of science shows that revolutions often come not from centralized initiatives but from distributed, bottom-up processes where individual investigators are free to follow their intuition and curiosity rather than predetermined milestones and deliverables. The rapid development of mRNA vaccines, for example, drew on decades of fundamental research into lipid nanoparticles, mRNA biology and immunology, which was initially dismissed as impractical and without immediate utility. This long gestation was often sustained against prevailing funding trends. When the COVID-19 crisis came, it was basic science not applied tinkering, that offered the lifeline (Dolgin, 2021).

There is a dangerous complacency in the belief that we will always have access to the next discovery just when we need it. Liu Cixin’s *The Three-Body Problem* dramatizes this fallacy in reverse: halting fundamental research becomes a weapon to cripple humanity (Liu, 2008). In our world, it seems that we are doing it to ourselves, voluntarily. The consequences will not be cinematic or immediate, but cumulative and corrosive: a slow erosion of the capacity to surprise ourselves. We will wake up not in a future of flying cars and fusion reactors, but in one where the limits of what we know become the limits of what we can do. Consider the example of the human genome: despite fully mapping it over two decades ago, we still only understand the function of a small fraction of it. The data is there, the knowledge is not. Without it, without new conceptual frameworks, we might never attain the wisdom to fulfill the initially inflated ambitions of saving patient lives. Our predicament cannot be captured more eloquently than by T.S. Eliot’s lament, “Where is the wisdom we have lost in knowledge? Where is the knowledge we have lost in information?” (Eliot, 1934).

There is a dangerous complacency in the belief that we will always have access to the next discovery just when we need it.

## Dreams of immortality

In recent years, the global scientific enterprise has witnessed a growing influx of private capital into fundamental research. The proliferation of private biomedical institutes, often named after tech billionaires, and the grand visions they project of curing all diseases, reversing ageing or colonizing Mars, have captured public imagination. The entry of enterprising parties into this space is not, in itself, detrimental or undesirable. Private sector involvement can complement public investment, and industry partnerships can accelerate translational pipelines. However, when governments begin to interpret such support as a substitute rather than a supplement – as is currently happening in several European countries and elsewhere – the risk becomes existential.

The history of industrial research laboratories illustrates both the potential and the limitations of private sector-sponsored research. The great corporate research laboratories of the 20^th^ century, Bell Labs, IBM Research, Xerox PARC, General Electric Research Laboratory, and others, contributed fundamental discoveries that transformed entire industries. Bell Labs alone was involved in the development of the laser, the transistor, information theory, the UNIX operating system, the discovery of the cosmic microwave background radiation, and numerous other breakthroughs. Yet, even these legendary institutions operated under special circumstances of minimal market competition that no longer exist. They also enjoyed the privilege of being embedded at the heart of a vibrant, publicly funded ecosystem of universities and research centres. As competitive pressures intensified and financial markets demanded shorter payback periods, these research laboratories gradually mutated into deployment-focused facilities that optimize existing technologies rather than creating new ones.

The pharmaceutical industry provides another case in point. Despite massive investments in research and development, pharmaceutical companies increasingly focus on “me-too” drugs that offer incremental improvements to existing medications, rather than seeking genuinely transformative treatments, based on new biological understanding. This focus reflects rational economic calculation: developing variations of existing drugs is less risky and more profitable than pursuing fundamental research into disease mechanisms.

The dreams of “breaking the spell of ageing” and defeating death, terraforming other planets or simulating consciousness are not, in themselves, unworthy. They can inspire and galvanize. But they should not become the organizing principles of scientific investment. When research funding is volatile or predicated on the whims of donors, entire scientific fields can wither. And when basic research is seen merely as a prelude to application rather than a value in itself, we risk hollowing out the intellectual capital that future generations will depend on. By concentrating resources on specific, headline-grabbing objectives, private initiatives risk distorting research priorities away from the open-ended, often obscure investigations that are the true engines of progress. Moreover, the privatization of basic research raises critical issues of equity, access, and accountability. Research agendas set by individuals, no matter how visionary, inevitably reflect their own values, biases, strategic interests, or escapism fantasies. The public, in turn, loses both voice and visibility in shaping the scientific frontier.

## The folly of abdicating public stewardship of basic research

The intrinsically ephemeral nature of private endeavors notwithstanding, investment in fundamental science by wealthy patrons appears to be a welcome supplement to strained public funding. Billion-dollar labs have emerged with often exaggerated promises of tackling major health challenges or extending life indefinitely. But this apparent renaissance conceals a deep misapprehension of what basic science is, how it is practiced, and why it cannot be delegated wholesale to the private sector (Stephan, 2012). Science in its most transformative form is a public good: unpredictable in outcome, collective in benefit and rooted in questions rather than applications. It requires boundless horizons and a tolerance for ambiguity that few private actors, compelled by expectation or image, can afford.

Moreover, the increasing reliance on the private sector distorts public understanding of what science can do. The belief in on-demand breakthroughs, cultivated by the techno-messianic ambitions of certain private institutions, leads to unrealistic expectations about the pace and nature of progress. It fuels pessimistic disillusionment when breakthroughs do not arrive, and distracts from the step-by-step, collective, and often understated work that underpins real discovery. It also masks the social dimension of science: its rootedness in shared infrastructure, open data, and communal norms of scrutiny and debate. These features cannot be purchased or programmed; they must be cultivated and protected through public institutions. Even when such top-down, mega projects produce valuable knowledge, they risk reinforcing a model of science as commodified performance; shiny, premeditated, and elite. This narrows the range of epistemic inquiry and erodes the foundational commitment to scientific pluralism and openness.

The dangers of privatizing the scientific enterprise are not only philosophical but also practical. Consider the study of *Caenorhabditis elegans*, the tiny nematode worm that has transformed our understanding of animal development, cell biology, ageing, and neuronal function. Few outside the public academic sphere would think to invest in a decades-long exploration of a transparent worm. Yet it was in this humble organism that researchers uncovered key principles of cell death and RNA interference, discoveries that won Nobel Prizes and paved the way for new therapies and technologies. These breakthroughs were made not because they were sought by design, but because a few scientists, supported by public grants, followed their curiosity. However, when governments use private sector involvement as a rationale to withdraw from funding curiosity-driven work, or to concentrate their support on translational and commercially relevant outcomes, they abdicate a responsibility that only the public sector can bear. Governments are the only actors with the mandate, the scale and the democratic accountability to invest in science that has no obvious application, no near-term payoff and no high-profile champions (Mazzucato, 2013). The lesson is simple if unglamorous: the most profound advances often begin not in grand visions but in a worm wriggling under a microscope, unassuming, unloved by markets, yet indispensable to science and progress.

The lesson is simple if unglamorous: the most profound advances often begin not in grand visions but in a worm wriggling under a microscope, unassuming, unloved by markets, yet, indispensable to science and progress.

Meeting the great challenges of our time requires not only technological ingenuity but also a robust, independent, and resilient scientific base. That base must be built not on the caprice of markets or magnates, but on the sovereignty and rock-solid commitment of public institutions to pursue understanding of fundamentals, however slowly it may unfold. To sustain a vibrant scientific culture, one that serves not only markets but humanity, governments must recommit to the support of basic research, not as a luxury, not as a hedge against private disinterest, but as a moral and civic obligation. The physical world does not yield to wishful aspirations or capital alone. It demands humility, persistence, and often decades of painstaking inquiry. To offload this responsibility is to risk a future shaped not by knowledge and wisdom, but by perilous hubris and narrow interests masquerading as vision.

A civilization that, with a conceited sense of entitlement, consumes its inheritance without renewing it, is one in decline. If we do not reverse course, if we do not restore funding, prestige and priority to frontier science, we will not accelerate into the future. We will plunge into decadence or, at best, stall in the precarious present.

A civilization that, with a conceited sense of entitlement, consumes its inheritance without renewing it, is one in decline.

## Supplementary information

Peer Review File
