# Supplementary material for: For the love of frontier research, or why Elon’s rockets keep blowing up
Source: EMBO Rep. 2025 Sep 29;26(20):4889–92. doi: 10.1038/s44319-025-00587-2 (PMC12549840; doi:10.1038/s44319-025-00587-2)
Supplement: Supplementary file 1 — Peer Review File [file 44319_2025_587_MOESM1_ESM.pdf]

EMBO reports

For the love of frontier research, or why Elon's rockets keep blowing up

Nektarios Tavernarakis

Corresponding author(s): Nektarios Tavernarakis (tavernarakis@imbb.forth.gr)

Review Timeline:

Submission Date:	17th Sep 25
Accepted:	18th Sep 25

Editor: Holger Breithaupt

Transaction Report: This article type is not peer reviewed.
